# Diagnostics and rehabilitation of post-stroke visual field loss using innovative visual field evaluation. The impact of losing driving privileges (DRIVE-study): Protocol for a randomized controlled trial

**DOI:** 10.1016/j.conctc.2026.101670

**Published:** 2026-07-21

**Authors:** Marte F. Rosenvinge, Per O. Lundmark, Stig Einride Larsen, Grethe Eilertsen, Helle K. Falkenberg

**Affiliations:** a National Centre for Optics, Vision and Eye Care, Faculty of Health and Social Sciences, University of South-Eastern Norway, Kongsberg, Norway; b Meddoc Research AS, Skjetten, Norway; c USN Research Group of Older Peoples’ Health, Faculty of Health and Social Sciences, University of South-Eastern Norway, Drammen, Norway

**Keywords:** Stroke, Vision rehabilitation, Vision impairments, Randomized controlled trial, Functional vision, VR-Technology, Pre-post study, Protocol

## Abstract

**Introduction:**

The overall aim is to explore the impact of losing driving privileges and to estimate the effect of compensatory vision rehabilitation in Norwegian stroke survivors whose driver's licenses have been revoked due to VFL. Specifically, to evaluate the effect of vision rehabilitation on perceived and measured functional vision, and secondarily explore markers for perceived functional vision and functional visual field that may predict an effect of vision rehabilitation.

**Methods:**

This study is an open, randomized controlled trial (RCT) with delayed-start design. Stroke survivors aged 20-85 years (N = 52) will be recruited from a vision rehabilitation clinic in Norway and randomized into two arms: A) immediate start of eight-week home-based vision rehabilitation intervention and B) delayed start (eight weeks) of the same eight-week vision rehabilitation intervention. A quantitative and qualitative approach will be applied to assess changes in functional vision after eight weeks of vision rehabilitation, and at twelve weeks post-intervention in both groups. The quantitative analysis includes descriptive statistics, plots and inferential statistics of effects. In addition, ten participants will be recruited for qualitative individual interviews before and after the eight-week intervention to explore experiences of living with VFL and participating in the intervention. For the qualitative analysis, we will apply thematic analysis.

**Conclusions:**

The study is designed as an RCT that will give important insight into the effectiveness of vision rehabilitation for visual field loss after stroke on functional vision measured by a novel VR-vision test.

**Trial registration:**

ClinicalTrials.gov: NCT07147660.

## 1 Introduction

Stroke is a leading cause of acquired visual field loss (VFL) in adults. Each year, approximately 12,000 people suffer a stroke in Norway [1], and more than 60% experience post-stroke visual impairments, including VFL [2]. The Norwegian NOR-OCCIP study found VFL in 80% of patients two weeks post-stroke and in 55% after six months [3]. VFL significantly affects quality of life and daily functioning, including reading, driving, work, communication, and mobility [[4], [5], [6], [7], [8], [9], [10], [11]]. Revoked driving privileges due to VFL are a major concern for stroke survivors and are often associated with reduced independence, depression, and lower quality of life [8,[12], [13], [14]].

European and Norwegian stroke care guidelines recommend compensatory strategies for VFL [15,16], which may improve reading speed, daily activities, and overall rehabilitation outcomes [[17], [18], [19]]. Evidence suggests that systematic eye movement and search training can enhance saccadic efficiency and exploration, improving functional vision and subjective daily living [13,[20], [21], [22], [23]]. However, few patients can adopt these strategies independently [24], and vision rehabilitation is not routinely offered in Norway after stroke [11,25] due to low sensitivity of clinical tests, lack of standardized protocols, and limited knowledge about its effectiveness.

Standard visual field tests are static and do not assess functional vision or a patient's ability to compensate for VFL, creating uncertainty about fitness to drive [[26], [27], [28]]. Functional visual fields reflect how visual information is processed and utilized under dynamic, attention-demanding conditions. Such measurements can capture dimensions including the integration of peripheral vision, motion detection, and divided attention required for safe navigation in complex traffic environments [29,30]. Although functional visual field measures are expected to be more representative of real-world driving than static perimetry, no methods have yet been specifically developed for this purpose. However, eye movement perimetry provides several promising alternative objective metrics such as gaze behavior and saccadic reaction times, which may serve as proxies for functional visual field assessment [31,32].

With an expected doubling of the population aged over 70 years by 2050, the number of individuals with VFL will increase substantially, creating a growing need for effective and accessible vision rehabilitation. Few studies have examined whether rehabilitation improves compensatory ability or whether new parameters in functional vision tests can be identified to measure this effect. Innovative solutions are therefore needed both to identify patients who can benefit most from rehabilitation and to assess compensatory ability in functional tasks such as driving.

### 1.1 Aim and research questions

*The overall aim* is to explore the impact of losing driving privileges and to estimate the effect of compensatory vision rehabilitation in Norwegian stroke survivors whose driver's licenses have been revoked due to VFL. Specifically, to evaluate the effect of vision rehabilitation on perceived and measured functional vision. *Secondarily*, to explore markers that predict an effect of vision rehabilitation on perceived and measured functional vision. To address these aims, we will conduct a randomized controlled trial (RCT) with delayed-start design in three sub-studies with distinct aims and research questions (RQs).

**Aim of sub-study I:** To investigate stroke survivors’ perceptions of visual functions and the influence of vision in daily activities and traffic situations. Furthermore, we aim to examine the relationship between perceived functional visual field and objectively measured visual field using standard clinical methods and VR-based assessments. **RQ1:** How do stroke survivors with VFL perceive their visual function and its impact on everyday life and traffic situations, and how is this related to patient-reported outcomes such as quality of life, fatigue, and self-regulation? **RQ2:** What factors influence the relationship between clinically measured visual field and perceived functional visual field?

**Aim of sub-study II:** To estimate the effects and outcomes of vision rehabilitation (the ability to compensate for VFL) in relation to visual functions as well as the ability to compensate for VFL in daily life. **RQ3:** How does vision rehabilitation affect measurements in terms of quality of life, self-regulation and motivation, fatigue and daily activities, visual functions including visual field measured with standard clinical methods and functional field measured with VR technology at the completion of rehabilitation? **RQ4:** How does vision rehabilitation affect measurements of quality of life, self-regulation and motivation, fatigue and daily activities, visual functions including visual field measured with standard clinical methods, and functional field measured with VR-technology at 12 weeks post-intervention? **RQ5:** What are the associations between baseline measurements and outcomes of vision rehabilitation for perceived and measured functional vision?

**Aim of sub-study III:** To explore experiences of living with VFL, losing driving privileges and the effect of vision rehabilitation on the ability to compensate for visual field loss in daily life and traffic situations. **RQ6:** How do people with stroke experience living with visual field loss, and how do they describe their strategies to compensate for their VFL in daily activities and traffic situations, before and after vision rehabilitation? **RQ7:** What are the benefits and drawbacks of participating in a home-based eight-week vision rehabilitation?

## 2 Methods

### 2.1 Study design

**Fig. 1:**
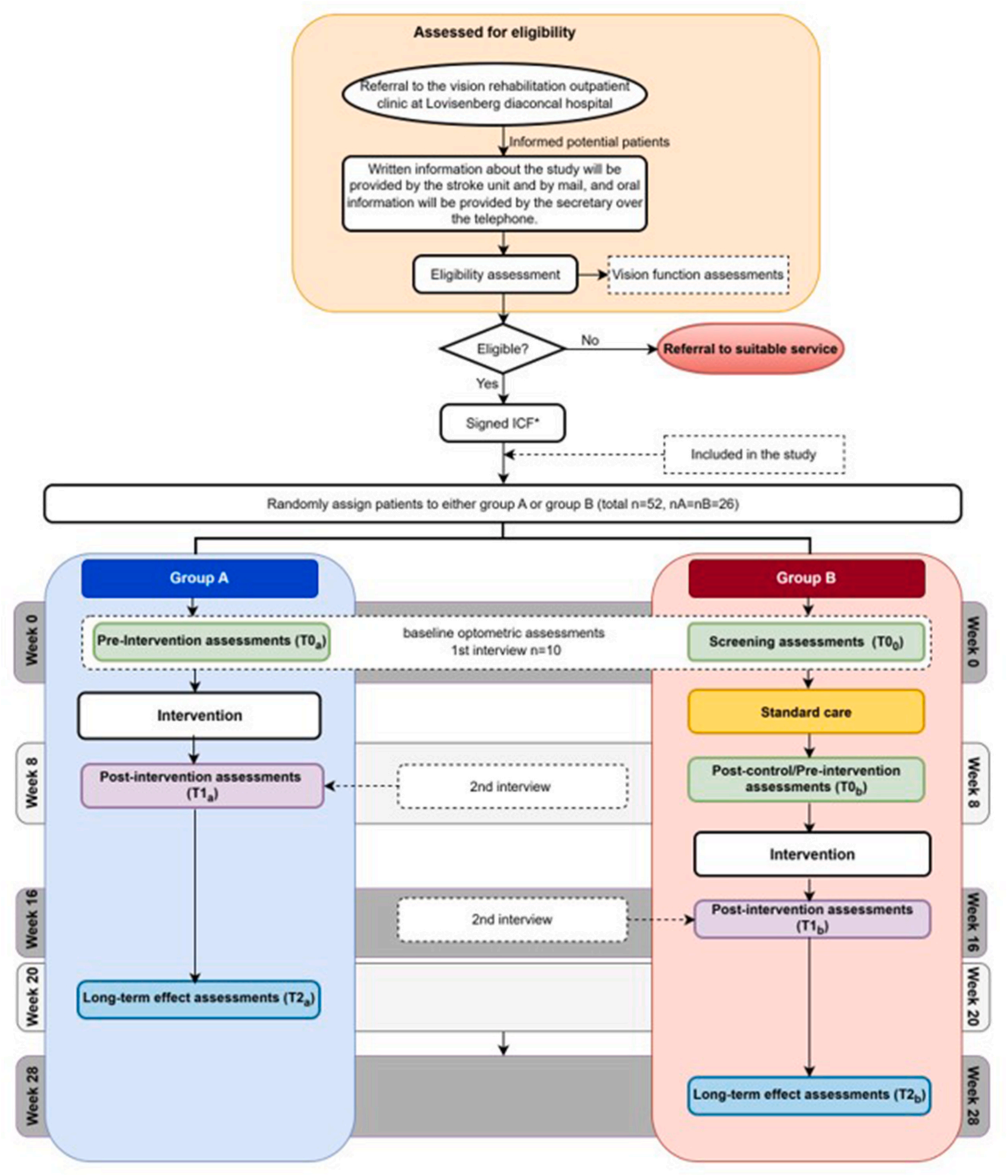
Flowchart of study recruitment, randomization and study procedure. Blue color represents the study procedure for group A (immediate) and red colour represents study procedure for group B (delayed). Green boxes represent pre-intervention (baseline) assessments for both groups; white color represents the intervention for both groups, light blue boxes represent long-term effect assessments for both groups. After randomization, dashed boxes indicate time points for the qualitative interviews.

The study will be performed as an open controlled and randomized stratified trial with a delayed-start design (Fig. 1). Participants will be randomized (1:1) to either an immediate (group A) or a delayed (group B) eight-week intervention. Both groups will perform objective and subjective assessments such as visual function assessments and questionnaires. Assessments will be performed at baseline, immediately after the intervention, and 12 weeks after the intervention. Participants assigned to the delayed group will begin the intervention with an eight-week delay.

### 2.2 Setting and recruitment procedures

Males and females aged 20 to 85 years with post-stroke VFL will be eligible for inclusion. Recruitment will be conducted continuously. Stroke survivors with VFL will be identified either in the acute phase in stroke units or at outpatient clinics at hospitals in South-Eastern Norway. Participants will not start the intervention until at least four weeks post-stroke to avoid the most sensitive period of natural spontaneous recovery. Potential participants will receive both oral and written information about the study at the point of care and will be referred to an outpatient vision rehabilitation clinic. They will be sent an information letter by mail and subsequently contacted by a secretary who will provide further details. This process ensures that participants have sufficient time to consider their participation. Written consent will be obtained by a secretary at the first consultation at the outpatient vision rehabilitation clinic (Fig. 1).

Information about the study will also be shared through patient organizations and networks, including The Norwegian Association of the Blind and Partially Sighted, the National Association for Heart, Lung and Stroke Patients, t he Norwegian Association of Stroke Survivors, and the Norwegian Vision in Stroke Network (NorVIS), as well as through social media platforms. The main researcher will be available to answer questions at any time.

### 2.3 Participant eligibility

All three sub-studies will use the same study sample. *Inclusion criteria*: Males and females between 20 and 85 years who do not meet the requirements for driving due to post-stroke VFL. *Exclusion criteria*: not having held a Norwegian Class 1 driver's license or driven a car within the last five years; not able to speak a Scandinavian language; not able to sign informed written consent; not willing to participate in all study activities throughout the intervention and follow-up; no technical proficiency or access to a PC (for online vision exercises); not able to attend data collection sessions in the region of the outpatient vision rehabilitation clinic; strabismus and/or neglect; eye diseases or other health conditions that disqualify them from meeting the health requirements for a Norwegian Class 1 driver's license; cognitive impairment/dementia or a severe psychiatric diagnosis.

People who do not meet the inclusion criteria will be offered ordinary vision rehabilitation at the outpatient vision rehabilitation clinic. A purposive sample of ten participants will be recruited from the same study population as included in sub-studies I and II. Participants will be invited to take part in two individual qualitative interviews before and after vision rehabilitation. Efforts will be made to ensure variation in age, sex and place of residence, to obtain a broad understanding of participants’ experiences of living with VFL and the consequences of revoked driving privileges for daily life.

### 2.4 Power analysis and sample size determination

A sample size power calculation was performed prior to the start of the study [33]. The power calculation serves as a lower-bound estimate of power if each patient has at least two measurements. Multiple observations per subject will increase the power and eliminate the impact of possible missing observations. With a significance level of 5%, a power of 90% and a Clinically Relevant Difference (CRD) of 1 x SD between groups A and B, at least 23 participants will be required in each group, with a total number of 46 participants. A total of 26 participants per group will be recruited to account for a 15% dropout, while retaining sufficient statistical power.

### 2.5 Randomization, stratification and blinding

**Fig. 2:**
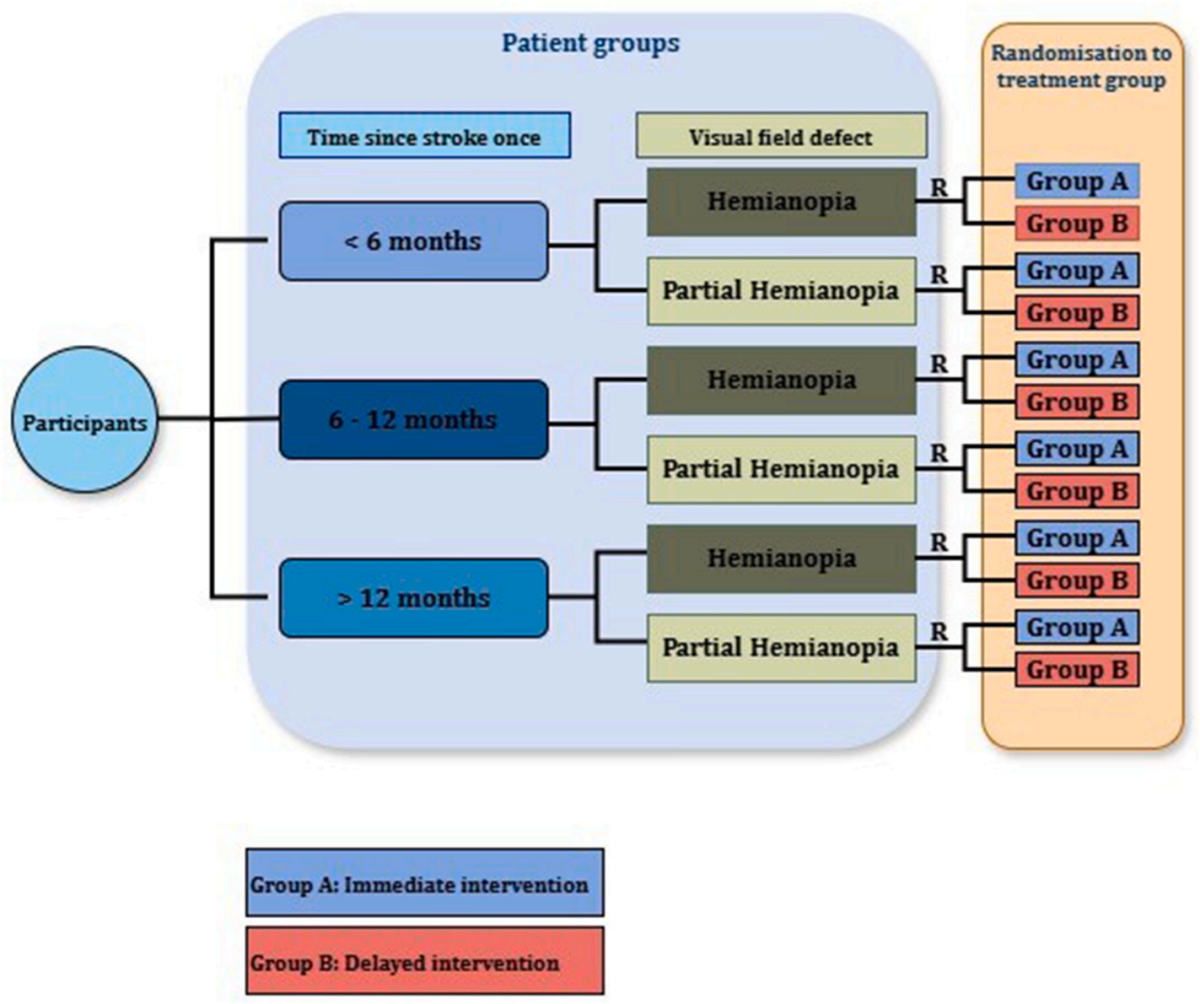
Study randomization and stratification factors.

“Time since stroke onset” and “Extent of visual field loss” are used as stratification factors. The first factor is divided into “< 6 months”, “6-12 months” and “> 12 months”, whereas the second is devided into complete- and partial hemianopia (Fig. 2). We define complete hemianopia as loss of an entire hemifield, while partial hemianopia refers to incomplete loss with preserved areas within the affected hemifield.

Within each strata the participants will be randomly allocated (1:1) to either immediate or delayed intervention at baseline. Allocation will be concealed in sealed envelopes prepared by an independent researcher. The main researcher will not gain access to the pre-randomization list.

Due to the nature of the intervention, neither participants nor the main researcher (MFR) can be blinded after allocation. Other project group members and researchers will be blinded to the treatment allocation.

### 2.6 The study intervention

After the baseline assessment, including an optometric assessment and completion of questionnaires (Table 1), participants will be randomly allocated to one of two groups (Fig. 1).

**Table 1:** Overview of data assessment schedule for SUB-STUDY I, II and III.

Sub-study	Name of test	Test information/measure of	Assessor	Time of assessment (week)				
				0	8	16	20	28^a^
**Background and clinical information**								
I	Anamnesis	Demographics	Researcher/participant	X				
I	MoCA	Cognitive functioning	Researcher	X				
I	NIHSS	Stroke severity	From patient record	X				
I	Heart cancellation	Visual attention	Researcher	X				
I	Barthel ADL-index	ADL-functions	Participant	X	X	X	X	X
**Self-reported questionnaires/interview**								
I&II	FSS	Fatigue severity	Participant	X	X	X	X	X
I&II	SRSI	Self-regulation, motivation	Researcher	X	X	X	X	X
I&II	BIVI-IQ	Visual impairment impact	Participant	X	X	X	X	X
I&II	EQ-5D-5L	HQOL	Participant	X	X	X	X	X
I&II	COPM	Occupational performance	Researcher	X	X	X	X	X
I&II	IDDL	Impact of driving on daily life	Participant	X	X	X	X	X
**Optometric vision assessments**								
I	LogMAR	Visual acuity	Optometrist/researcher	X	X	X	X	X
I	Cover-test	Heterophoria	Optometrist/researcher	X	X^a^			
I	MARS-test	Contrast sensitivity	Optometrist/researcher	X	X	X	X	X
I	Near point of convergence	Convergence	Optometrist/researcher	X	X	X	X	X
I	HRR	Colour vision	Optometrist/researcher	X	X^a^			
I	TNO	Stereoacuity	Optometrist/researcher	X	X	X	X	X
**Visual Field Assessments**								
I&II	Esterman	Binocular VF	Researcher	X	X	X	X	X
**Primary outcome**								
I&II	BCAM VFT	Visual field test	Researcher	X	X	X	X	X
**Secondary outcome**								
I&II	IReST	Reading speed	Researcher	X	X	X	X	X
I&II	BCAM Saccade test	Saccade latency, peak velocity, accuracy	Researcher	X	X	X	X	X
**Qualitative method**								
III	Individual interviews	Experiences	Researcher/participant	X	X	X		

a Only for group B.

Immediate group: Participants in the immediate group will receive the intervention immediately. The intervention consists of eight weeks of home-based training exercises for visual scanning, visual search and eye movements, aimed at developing compensatory strategies for visual field loss. The training will include both a licensed online vision training program (Vision Builder) [34] and manual exercises. Participants will be instructed to complete 20 min of vision training daily, with a minimum recommended total training of 20 h over the eight-week period.

Adherence, motivation and perceived visual function will be closely monitored throughout the intervention. Each participant will receive a short weekly telephone check-in with open-ended questions, and they will be asked to maintain a training diary. Adherence will be categorized as “good”, defined as performing vision training 5–7 days/week, as “partial”, performing vision training 1–3 days/week, or “none”, as no training. Active measures will be taken to promote compliance. Visual function assessments and questionnaires will be repeated immediately after the intervention in week eight, with a final follow-up assessment conducted 12 weeks after completion. Additionally, participants will evaluate the acceptability and relevance of the training through a survey and qualitative interviews.

Delayed group: Participants in the delayed-start group will serve as a waiting-list control group and receive standard care for the first eight weeks. After the waiting-list control period and corresponding assessments, they will receive the same vision rehabilitation intervention as described for the immediate group.

### 2.7 Study procedure

#### 2.7.1 Data collection

All data collection will be conducted by the main researcher (MFR). An authorized optometrist will assist with the optometric assessment data collection. All vision-related assessment data will be collected at a local hospital in Norway. Data collection, including vision assessments and questionnaires, will take place at three or four time points, depending on group allocation (Fig. 1). Baseline evaluations include background demographics and vision function and cognitive assessment. Table 1 shows a detailed description of the assessment schedule for the three sub-studies.

#### 2.7.2 Data handling

The electronic data capture (EDC) system InCRF will be used as the study database. Paper-based Case Record Forms (pCRFs) will be developed as templates to serve as the framework and reference for database development and to collect questionnaire information.

Data will be entered into electronic CRFs (eCRFs) in the InCRF study database in two ways.

• On-site manual data entry from pCRFs and questionnaires
• External data transfer for technical data from BulbiCAM examinations

Questionnaire data will be entered into the study database by MFR. Technical data from BulbiCAM examinations will be exported as encrypted files and subsequently imported into InCRF by a data manager (DM). Prior to study start, a data entry instruction (DEI) document will be made.

Participant identities in the database will be anonymized. Each participant will be assigned a unique study identification number, which will be stored in the database. The DM will be responsible for data validation of the eCRFs. In cases of missing data, logical inconsistencies, or interpretation issues, an electronic query (eQuery) will be issued through InCRF. Initial data validation will be performed by the DM once data from the first participant have been entered into InCRF. When all required participants have completed their final visit and the data entry have been completed in InCRF, the DM will perform the final data verification checks.

Screening analyses for logical errors will be performed on an ongoing basis, and any identified errors will be corrected after updated information is obtained from the site. When all detected errors have been resolved, the primary study database will be locked. The database will then be converted into a labeled SAS dataset, which will also be locked to prevent any changes or additions. In this SAS copy, the responsible statistician may create derived variables but may not correct or otherwise alter the original data.

If data corrections are required after database lock, the primary study database must be reopened and corrected in accordance with standard international procedures for database changes and documentation.

### 2.8 Statistical methods

All participants fulfilling the inclusion and exclusion criteria, have given informed consent to participate and have started BCAM registration, are classified as included in the study. The participants classified as dropouts will be excluded from the statistical analysis. Descriptive analyses will be used to summarize the key characteristics of the participants. Categorical variables will be presented as frequencies and percentages, while means and standard deviations will be calculated for continuous and ordinal data. Randomization will be stratified by time since stroke onset and visual field loss using block randomization with varying block sizes, and these stratification factors will be accounted for in the analysis. Differences in age and sex will be considered in the analysis.

Continuously distributed variables will be presented as mean value with 95% confidence intervals. Standard deviation (SD) will be used as an expression of dispersion. Categorized variables will be given in classical contingency tables. Categorical variables will be analyzed by contingency table analysis. Intervention effects will be analyzed using ANOVA with repeated measurements. Depending on the initial analyses of the anonymized data, additional in-depth statistical analyses may be conducted to explore relationships between selected variables. Where appropriate, correlation and regression analyses will be performed. Data will be processed and analyzed using SAS software. For analysis of the qualitative data, as in sub-study III, all interviews will be audio-recorded, transcribed verbatim, and analyzed using thematic analysis according to Braun & Clarke [35].

### 2.9 Outcome measures

The primary outcome is the change in the Saccadic Reaction Time (SRT), measured using the BulbiCAM visual field test [36]. SRT will be assessed at all measurement points throughout the study to evaluate both immediate and sustained effects of the vision rehabilitation training. Secondary outcomes include changes in occupational performance and satisfaction using the Canadian Occupational Performance Measure (COPM) [37,38], health-related quality of life (EQ-5D-5L) [39], Self-regulation Skills Interview [40], Brain Injury Associated Visual Impairment Impact Questionnaire (BIVI-IQ) [41], and a questionnaire on the impact of driving on daily life (IDDL). All will be evaluated at each measurement point. Additionally, changes in reading speed will be measured using the Norwegian version of the International Reading Speed Texts (IReST) [42] and visual functions will be assessed. In addition to these quantitative outcomes, qualitative data will be collected through semi-structured interviews to explore participants' experiences with vision loss, the impact of losing their driver's license on daily life and quality of life, and their expectations of vision rehabilitation before starting the home-based training and their perceptions of the vision rehabilitation after the eight weeks of training.

### 2.10 Organization of data collection and data management

Data will be managed as recommended by the Norwegian Agency for Shared Services in Education and Research. Participants’ personal information will be stored on one of the devices that only the main researcher has exclusive access to. This computer is a separate PC where no other data is stored. All data from this test will be pseudoanonymized before use. Data recorded in the BulbiCAM will be registered with the participants project ID-numbers. All data from the electronic devices will be exported from the software to the InCRF database by an encrypted IRON-KEY. Data from the interviews will be collected by audio recording through either a digital platform (Zoom/Microsoft Teams) or physically with a Dictaphone through Nettskjema (nettskjema.uio.no) at a location chosen by the participant. Recordings of the interviews, transcription files and all other data will be stored pseudoanonymized on a research server. Paper-registered data will be temporarily stored in a locked cabin at the hospital research facility before being stored digitally on the research server. Data collection, breaks and the number of sessions, will be tailored to the individual participant to minimize fatigue.

### 2.11 Discontinuation/withdrawal

A participant may discontinue the study or withdraw their consent at any time without concerns about their future treatment or care. If a participant does not attend an agreed assessment/meeting, the researcher should try to motivate the participant to continue. If a participant decides not to continue for reasons documented as not being related to the research question, they will be classified as “dropouts” and replaced by a new participant. Reasons for discontinuation will be discussed in a dialogue between the lead researcher and the participant and subsequently evaluated by the research team in relation to the research questions. The dropouts will not be included in the statistical analysis. Participants who discontinue or exit the study for reasons related to the study question or to the study procedure or equipment will be classified as “patient withdrawals”. These will not be replaced, but they will be included in the analysis using a last observation-carried-forward procedure.

### 2.12 Ethical considerations and regulatory approvals

The study will be performed in accordance with the tenets of the Declration of Helsinki [43] and the latest amendments to Good Clinical Practice (GCP). The study has approval from the Regional Committees for Medical and Health Research Ethics South East Norway (ref: 781461) and the Norwegian Agency for Shared Services in Education and Research (ref: 457711). The study is registered as a clinical trial at ClinicalTrials.gov (ref: NCT07147660). Publication of study results will be according to open access policy. Adverse effects will be noted and reported according to clinical guidelines. The project is also approved by the data protection officer at the hospital for data collection.

### 2.13 Patient group and reference group involvement

The study design and objectives were developed by the researchers in collaboration with the user representatives and an expert vision rehabilitation group. The reference groups the National Association for Heart, Lung and Stroke Patients, Norwegian Association for Stroke Survivors and Norwegian Association for the Blind and Partially Sighted contributed actively to the planning of the study, piloting and recruitment, and will help with dissemination of results.

## 3 Discussion

Visual field loss (VFL) after stroke is common and has major implications for independence and safety, particularly in relation to driving. Early identification and rehabilitation of VFL are recommended in national and international guidelines [15,16], yet these measures are not routinely implemented in clinical practice in Norway [11]. This gap is partly understandable: traditional visual field assessments are static and resource-intensive. Another major contributing factor is the lack of structured vision screening, such as Competence, Rehabilitation of Sight after Stroke (CROSS) [44], and the limited availability of objective perimetry/visual field assessment, equipment and standardized rehabilitation programs. Furthermore, compensatory strategies often require structured training and follow-up, which many healthcare services lack the capacity to provide.

The use of VR-based functional vision testing in this study addresses some of these challenges by offering a dynamic, performance-oriented approach that could be integrated into clinical workflows. Functional visual field testing is expected to provide new insights into the mechanisms that help compensate for visual field loss in traffic situations and everyday life. However, implementation of such technology depends on access to equipment, digital competence among patients and clinicians, and clear evidence of clinical benefit. These considerations will be important for interpreting the feasibility and scalability of the intervention beyond the trial setting. The prevalence of visual field loss (VFL) after stroke is expected to rise significantly as the population ages, with projections indicating a doubling of the number of individuals over 70 years by 2050 [45]. This demographic shift will increase demand for vision rehabilitation services, yet current healthcare systems face resource constraints that make widespread in-person rehabilitation difficult to implement. Therefore, efficient and sustainable solutions are essential.

Home-based rehabilitation offers a promising approach to address these challenges by improving accessibility and reducing the burden on specialized centers [13,17]. If proven effective, home-based programs could allow more patients to receive timely intervention without requiring extensive travel or increased clinic capacity. Weekly follow-up calls during the intervention are a key feature to maintain motivation, answer questions, and mitigate dropout risk, which is often a concern in remote programs. This structured contact also supports adherence and provides an opportunity to address difficulties early. However, home-based rehabilitation is not without challenges. Variability in adherence, differences in home environments, and limited direct supervision may affect consistency and outcomes. Digital literacy and access to necessary equipment can also be barriers for some older adults. These factors must be considered when interpreting results and planning future implementation. If feasibility and effectiveness are demonstrated, home-based rehabilitation could represent a scalable solution to meet the growing need for vision rehabilitation in an aging population.

This study has several strengths. First, it addresses a significant gap in post-stroke care by employing a randomized controlled delayed-start design to evaluate the effect of compensatory vision rehabilitation in a real-world setting, using a home-based approach that reflects practical clinical applications. The integration of innovative VR technology for functional vision assessment is another strength, as it enables dynamic measurement of reaction times and eye movements, which traditional static tests cannot capture. Including qualitative interviews alongside quantitative measures will provide insight and a richer understanding of patient experiences and perceptions of compensations in daily life. Furthermore, the delayed-start design ensures that all participants receive the intervention, which is ethically sound and may improve recruitment and retention, and allows comparison with a waiting-list control. While blinding participants and the main researcher post-allocation is not possible, allocation concealment and standardized procedures will help reduce bias.

However, the study also has limitations. The inability to blind participants and the main researcher post-allocation may introduce bias, although allocation concealment and standardized procedures will help mitigate this. Adherence to home-based training may vary, potentially affecting intervention fidelity.

Despite these limitations, this study represents an important step toward developing accessible vision rehabilitation strategies and improving functional vision assessment for individuals with visual field loss after stroke. Findings will primarily inform on feasibility and provide preliminary evidence on whether home-based rehabilitation and VR-based assessment are promising approaches for future research and clinical practice.

## 4 Conclusion

This randomized controlled study will evaluate the effect of home-based compensatory vision rehabilitation on functional vision using innovative VR technology to measure functional visual field, reaction times, eye movements, and the impact of losing driving privileges. In addition, the study will provide new insights into how patients perceive and experience compensating for VFL in traffic situations and everyday life. If proven effective, this vision rehabilitation program could become an accessible and cost-effective intervention, improving independence, quality of life, and potentially influencing future guidelines for driving assessments and vision rehabilitation.

## Informed consents

Written informed consent will be obtained from the participants before inclusion in the study.

## Funding

Open access funding is provided by the University Of South-Eastern Norway, 10.13039/100015996USN. The research is conducted by employees at USN with no external funding.

## CRediT authorship contribution statement

**Marte F. Rosenvinge:** Conceptualization, Methodology, Project administration, Writing – original draft, Writing – review & editing. **Per O. Lundmark:** Conceptualization, Methodology, Supervision, Writing – review & editing. **Stig Einride Larsen:** Formal analysis, Methodology, Supervision, Writing – review & editing. **Grethe Eilertsen:** Conceptualization, Methodology, Supervision, Writing – review & editing. **Helle K. Falkenberg:** Conceptualization, Funding acquisition, Methodology, Project administration, Supervision, Writing – original draft, Writing – review & editing.

## Declaration of competing interest

The authors declare that they have no known competing financial interests or personal relationships that could have appeared to influence the work reported in this paper.

## Data Availability

No data was used for the research described in the article.
